# Entropy of radiation: the unseen side of light

**DOI:** 10.1038/s41598-017-01622-6

**Published:** 2017-05-10

**Authors:** Alfonso Delgado-Bonal

**Affiliations:** University of Salamanca, Institute of Fundamental Physics and Mathematics, Pza. de la Merced S/N, 37008 Salamanca, Spain

## Abstract

Despite the fact that 2015 was the international year of light, no mention was made of the fact that radiation contains entropy as well as energy, with different spectral distributions. Whereas the energy function has been vastly studied, the radiation entropy distribution has not been analysed at the same speed. The Mode of the energy distribution is well known –Wien’s law– and Planck’s law has been analytically integrated recently, but no similar advances have been made for the entropy. This paper focuses on the characterization of the entropy of radiation distribution from an statistical perspective, obtaining a Wien’s like law for the Mode and integrating the entropy for the Median and the Mean in polylogarithms, and calculating the Variance, Skewness and Kurtosis of the function. Once these features are known, the increasing importance of radiation entropy analysis is evidenced in three different interdisciplinary applications: defining and determining the second law Photosynthetically Active Radiation (PAR) region efficiency, measuring the entropy production in the Earth’s atmosphere, and showing how human vision evolution was driven by the entropy content in radiation.

## Introduction

Entropy is a quantity as fundamental as energy, nevertheless, the analysis of the entropy content in radiation is not fully exploited yet. Although it has been applied in engineering and science, its existence is generally unknown and there are still many faces to explore.

The analysis of radiation entropy has been mainly carried out in the context of optics, whereas in other fields such as atmospheric physics or statistics the function has been studied only occasionally. The research in radiation entropy was very present at the beginnings of the quantum theory until the seminar paper of Bose^1^, who showed how to obtain Planck’s law without the necessity of determining the entropy of radiation. Soon after Planck proposed his radiation law and the associated equation for the spectral entropy, von Laue^2^ analysed the phenomena of interference from a thermodynamical perspective, discussing the applicability of the second law to radiation and the additivity of the entropy. His ideas were mostly continued in the fields of coherence and polariazation, where the radiation entropy approach has proven to be very productive^3^. In that sense, the importance of the radiation entropy in the scattering process is a field in current development, and the ideas are masterly investigated in ref. 4.

Although the optical properties have been investigated, the statistical ones have not received much attention, as well as the possible applications of the magnitude. It is the intention of this manuscript to amend this situation, and here I survey the statistical properties of the entropy of radiation and underline some new applications, which have not been investigated before in the knowledge of the author.

Planck’s laws for energy and entropy are density functions which are characterized by their Mode, Median and Mean. The Mode of Planck’s function is determined by the Wien’s displacement law, and it is well known that the maxima occur at different positions depending on the choice of the spectral variable^5^. The situation for the entropy of radiation is quite the same, and it is possible to determine its maxima as a Wien’s like law, with a different values of the coefficients depending on the selected spectral variable. This coefficient is a constant which cannot be obtained from the energy distribution.

The integration of Planck’s law in the whole spectrum gives directly the Stefan-Botlzmann’s law with the widely known dependence on the fourth power of the temperature, and the integration of the entropy gives the equivalent law with the third power dependence on the temperature. Although the complete integration is well known, the somehow incommodious mathematical form of the entropy distribution makes it not very suitable for integration in a region. In this paper, I show how to obtain a polylogarithmic expression to determine the entropy fractional emissive power of blackbody radiation in a given spectral band, and by making use of this expression, the Median and the Mode of the distribution are determined. Besides these measures of central tendency, the most important measures of variability are studied in this manuscript, which are the Variance, Skewness and Kurtosis, and a general expression for the moment of order *n* is provided.

The field of entropy radiative transfer is not fully developed yet, but it is of the utmost importance in many different fields such as heat transfer by radiation, climate sciences or information theory, and the interest in this magnitude has increased recently. One of the issues that held back the analysis of the entropy of radiation was the lack of direct applications, and in this paper I show how the concept can be applied to three different fields.

By making use of the two laws of thermodynamics and the expressions of the energy and entropy of radiation, the maximum obtainable work from radiation can be determined, a magnitude which is called *exergy* ^6, 7^. This kind of analysis –named second law analysis– allows us to determine not only the amount of energy but also its quality, greatly important in industrial processes and, in particular, in solar energy conversion devices. Although the concept is currently being used, the determination of the exergy in spectral regions has been carried out by numerical procedures up to now. The analytical expression to determine the fractional emissive power for the exergy is derived here, which permits the analysis of the obtainable work from radiation in a determined band or spectral region more quickly and accurately. When the whole spectrum is considered, the expression is reduced to the well known Petela’s equation^8^.

One of the most important processes which makes use of radiation is photosynthesis. However, at the same time than the organism is obtaining energy from radiation, the organism is losing energy itself by emitting radiation, as a consequence of its own temperature. Thus, the exergy concept is more suitable to analyze the efficiency of the process, and the second law efficiency of the photosynthetic active radiation (PAR) region is defined and determined here using this formalism.

The spectral entropy law –which is the entropy of bosons– plays a role in the radiative transfer of entropy equivalent to the Planck’s law in classical radiative transfer. Even though the expression for the entropy of radiation used in this manuscript was proposed for the equilibrium situation, it has been proved that the equation holds for non-equilibrium situations^9–11^. By definition, blackbody radiation is that radiation which produces the largest amount of entropy for a given quantity of energy^12^. By characterizing the entropy content in blackbody radiation, it is possible to define more accurately the deviations from this kind of radiation.

In particular, the entropy expression can be applied to the analysis of radiation in the atmosphere. Here I present the entropy spectrum for Earth’s upwelling radiation, with the intention to show more applications of the entropy analysis. By comparison between the obtained values and those expected for a blackbody, the entropy production is characterized in a wavelength basis, showing that different absorption bands lead to different values of entropy production in the atmosphere. Therefore, the contribution of the different chemical species to the atmospheric entropy production budget can be inferred from this sort of analysis.

The entropy concept is applied in many disciplines besides engineering and science. In particular, information theory^13^ makes a very interesting use of the entropy magnitude, understanding it in relation with the information contained in a system. In a previous number of this journal^14^, I hypothesized that entropy plays a major role as driving force in the biological evolution of human eyesight, by determining the maximum of this function. Using the equations determined in this paper, I prove that the importance of the entropy in human vision is independent of the spectral variable^15^, opening new ways to understand human perception and inference.

Finally, some mathematical appendixes complete this paper with the required explanations and demonstrations, including a short introduction to the historical development of the field for the interested reader.

## The Mode: Wien’s law for the entropy of radiation

The expressions for energy (*L*) and entropy (*S*) of blackbody radiation in wavelength and frequency are:

1 $${L}_{\lambda }=\frac{2h{c}^{2}}{{\lambda }^{5}}\frac{1}{{e}^{\frac{hc}{k\lambda T}}-1}$$

2 $${S}_{\lambda }=\frac{2kc}{{\lambda }^{4}}[(1+\frac{{\lambda }^{5}{L}_{\lambda }}{2h{c}^{2}}){\rm{l}}{\rm{o}}{\rm{g}}(1+\frac{{\lambda }^{5}{L}_{\lambda }}{2h{c}^{2}})-\frac{{\lambda }^{5}{L}_{\lambda }}{2h{c}^{2}}{\rm{l}}{\rm{o}}{\rm{g}}(\frac{{\lambda }^{5}{L}_{\lambda }}{2h{c}^{2}})]$$

and

3 $${L}_{\nu }=\frac{2h{\nu }^{3}}{{c}^{2}}\frac{1}{{e}^{\frac{h\nu }{kT}}-1}$$

4 $${S}_{\nu }=\frac{k{\nu }^{2}}{{c}^{2}}[(1+\frac{1}{{e}^{\frac{h\nu }{kT}}-1}){\rm{l}}{\rm{o}}{\rm{g}}(1+\frac{1}{{e}^{\frac{h\nu }{kT}}-1})-\frac{1}{{e}^{\frac{h\nu }{kT}}-1}{\rm{l}}{\rm{o}}{\rm{g}}(\frac{1}{{e}^{\frac{h\nu }{kT}}-1})]$$

Expressions 1–4 were firstly obtained by Planck^16^, and were demonstrated later by many other ways^1, 17^, including non-equilibrium situations^9^.

**Figure 1:**
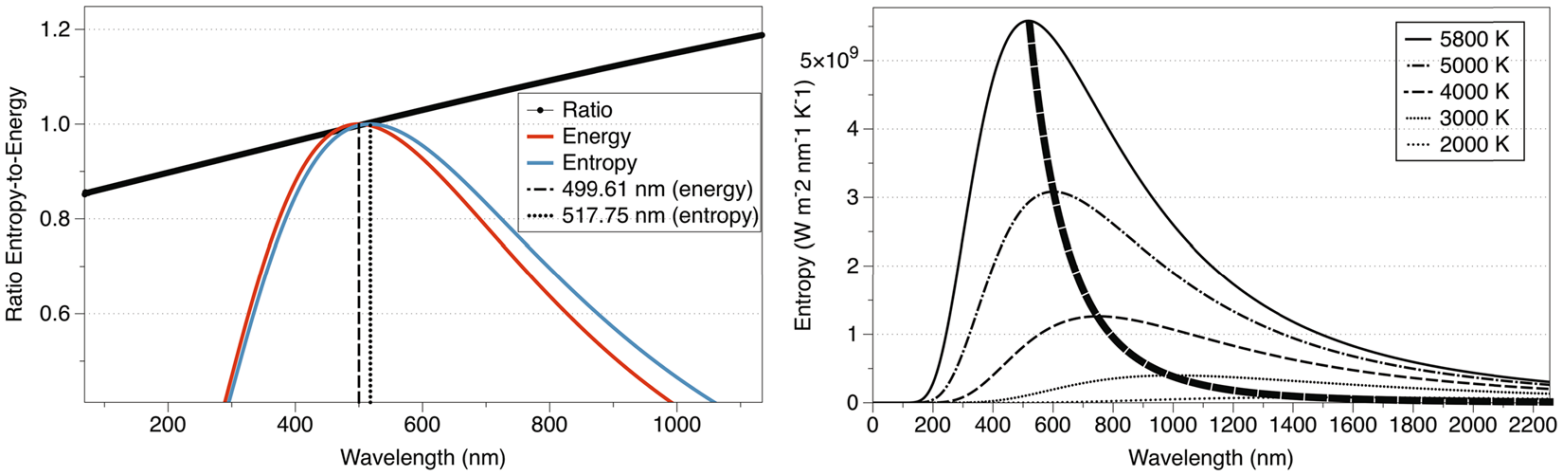
(**a**) (left): Normalized entropy (blue line) and energy (red line) of blackbody radiation at 5800 K. The ratio entropy-to-energy is determined by the black line. (**b**) (right): Entropy of blackbody radiation at different temperatures. The behaviour resembles the energy distribution.

Looking at the previous equations, it is clear that the entropy distribution is different than the energy one, but its spectral distribution has not been of much interest. Figure 1a represents the normalized spectra of blackbody energy and entropy, showing that the curves and the position of their maxima are different. The Wien’s displacement law which determines the wavelength of maximum energy emission as a function of the temperature of the blackbody does not apply for the entropy directly.

Using Equations 2 and 4, the statistical properties of the entropy of radiation can be analysed. The function is non-symmetric, it is always positive (because it is a density function), it has only one maximum, and the total integration gives the equivalent Stefan-Boltzmann’s law, *S* = 4/3*σT* ^3^. The wavelength corresponding to the maximum of the distribution depends on the temperature, as shown in Fig. 1b for different blackbody temperatures.

This behaviour resembles the energy distribution, and will be determined below by a Wien’s like law with a different coefficient. As in the case of the energy, the value of the constant (which determines the “position” of the maxima) will depend on the spectral variable used in the description of radiation, and it will be determined based on a parameter called dispersion rule coefficient, *m* ^18^.

Energy and entropy distribution laws are density functions and the exchange between spectral variables must be carried out using differential terms. In particular, the relation between wavelength and frequency is ${S}_{\lambda }(\lambda ,T)=-\,\frac{d\nu }{d\lambda }{S}_{\nu }(\nu (\lambda ),T)$, where $d\nu /d\lambda =-c/{\lambda }^{2}$. Generalizing this situation, it is possible to express the entropy function S_*ϑ*_ as a density distribution function defined differentially by:

5 $$d{W}_{\vartheta }={S}_{\vartheta }d\vartheta $$

where *W*_*ϑ*_ represents the entropy emissive power in the differential interval d*ϑ*. In this way it is clear that the emissive power is independent on the selected spectral variable, and the function S_*ϑ*_ represents the spectral entropy emissive power per unit physical quantity interval *ϑ* from a blackbody at absolute temperature *T*. The different choices of spectral variables, such as wavelength, frequency, squared wavelength, etc., are studied here in terms of the dispersion rules^19^.

The Mode of the distribution is obtained from the condition *dS*/*dϑ* = 0. When the analysis is performed in frequency, *dS*/*dν* = 0, the transcendental equation which determines the maximum of the distribution is (see Appendix A):

6 $$3x{({e}^{x}-1)}^{2}+3x({e}^{x}-1)-{x}^{2}{({e}^{x})}^{2}+2{\rm{l}}{\rm{o}}{\rm{g}}(\frac{1}{{e}^{x}-1}){({e}^{x}-1)}^{2}-x{e}^{x}({e}^{x}-1)=0$$

which can be numerically solved, obtaining the value of *x* = 2.538231893. Undoing the change of variable $x=\frac{h\nu }{kT}$, the peak is accomplished at:

7 $$\frac{{\nu }_{max}}{T}=5.28882\times {10}^{10}{K}^{-1}{s}^{-1}$$

In the case of the energy distribution, the maximum occurs at 5.87893 × 10^10^ *K* ^−1^ *s* ^−1^, a value slightly different.

Similarly, in the case of wavelength starting from Equation 2 and doing the change of variable $x=\frac{hc}{\lambda kT}$, the transcendental equation is ref. 14:

8 $$5x{({e}^{x}-1)}^{2}+5x({e}^{x}-1)-{x}^{2}{({e}^{x})}^{2}+4{\rm{l}}{\rm{o}}{\rm{g}}(\frac{1}{{e}^{x}-1}){({e}^{x}-1)}^{2}-x{e}^{x}({e}^{x}-1)=0$$

The solution of the equation is *x* = 4.7912673578 and, undoing the change of variable, the Wien’s entropy displacement law in wavelength looks like:

9 $$\lambda T={b}_{{\rm{entropy}}}=3.00292\times {10}^{-3}\,{\rm{m}}\,{\rm{K}}$$

different than the value of *b*_energy_ = 2.89777 × 10^−3^ m K.

Looking at Equations 6 and 8, it is easy to generalize the transcendental equation to any value of the dispersion coefficient, *m*. The general transcendental equation reads:

10 $$\begin{array}{c}m\cdot x{({e}^{x}-1)}^{2}+m\cdot x({e}^{x}-1)\,-{x}^{2}{({e}^{x})}^{2}+(m\,-\,1)\cdot {\rm{l}}{\rm{o}}{\rm{g}}(\frac{1}{{e}^{x}-1}){({e}^{x}-1)}^{2}-x{e}^{x}({e}^{x}-1)=0\end{array}$$

Which can be solved for each value of *m*. The values of the Wien’s coefficients are given in Table 1, along with the explanation of the dispersion rule used.

**Table 1:** Wien’s peaks for the energy and the entropy of radiation for different dispersion rules, corresponding to different values of the dispersion coefficient *m*.

*ϑ*	B_*ϑ*_ (T)d*ϑ*	Dispersion rule	*m*	Energy	Entropy
*ν* ^2^	$2\nu {B}_{{\nu }^{2}}(T)d\nu$	frequency-squared	2	$\frac{hc}{{k}_{B}\mathrm{(1.593624}\ldots )}$	$\frac{hc}{{k}_{B}\mathrm{(1.178179641}\ldots )}$
*ν*	*B*_*ν*_(*T*)*dν*	linear frequency	3	$\frac{hc}{{k}_{B}\mathrm{(2.821439}\ldots )}$	$\frac{hc}{{k}_{B}\mathrm{(2.538231893}\ldots )}$
$\sqrt{\nu }$	$\frac{1}{2\sqrt{\nu }}{B}_{\sqrt{\nu }}(T)d\nu$	square root frequency	7/2	$\frac{hc}{{k}_{B}\mathrm{(3.380946}\ldots )}$	$\frac{hc}{{k}_{B}\mathrm{(3.137016422}\ldots )}$
log *ν*	$\frac{1}{\nu }{B}_{\mathrm{log}\nu }(T)d\nu$	logarithmic frequency	4	$\frac{hc}{{k}_{B}\mathrm{(3.920690}\ldots )}$	$\frac{hc}{{k}_{B}\mathrm{(3.706085183}\ldots )}$
log *λ*	$\frac{1}{\lambda }{B}_{\mathrm{log}\lambda }(T)d\lambda$	logarithmic wavelength	4	$\frac{hc}{{k}_{B}\mathrm{(3.920690}\ldots )}$	$\frac{hc}{{k}_{B}\mathrm{(3.706085183}\ldots )}$
$\sqrt{\lambda }$	$\frac{1}{2\sqrt{\lambda }}{B}_{\sqrt{\lambda }}(T)d\lambda$	square root wavelength	9/2	$\frac{hc}{{k}_{B}\mathrm{(4.447304}\ldots )}$	$\frac{hc}{{k}_{B}\mathrm{(4.255382544}\ldots )}$
*λ*	*B*_*λ*_(*T*)*dλ*	linear wavelength	5	$\frac{hc}{{k}_{B}\mathrm{(4.965114}\ldots )}$	$\frac{hc}{{k}_{B}\mathrm{(4.791267357}\ldots )}$
*λ* ^2^	$2\lambda {B}_{{\lambda }^{2}}(T)d\lambda$	wavelength-squared	6	$\frac{hc}{{k}_{B}\mathrm{(5.984901}\ldots )}$	$\frac{hc}{{k}_{B}\mathrm{(5.838126229}\ldots )}$

## The Median: fractional emissive power of entropy

In the previous section, I characterized the wavelength of maximum emission of entropy by a blackbody emitter. Although the maximum of the spectral distribution of entropy is of critical importance, the knowledge of the entropy emissive power in a spectral region is required in the fields of radiative transfer, engineering and climate sciences, for example.

The determination of the fractional emissive power is usually done numerically, despite the fact that analytical expressions are tremendously advantageous in computing and provide an intrinsic elegance to the solution of the problem. In the field of thermal radiation, Planck’s equation is conventionally integrated using numerical procedures, albeit an analytical solution in the form of polylogarithms has appeared recently in the literature^20^. The definition and properties of polylogarithms are briefly reviewed in Appendix C.

Although less known, the entropy of radiation has a different radiative transfer equation^21, 22^ and the source function is a nonlinear complex function. By making use of polylogarithms, an analytical expression for the integration of the entropy of radiation is achieved here. Moreover, after straightforward arithmetical transformations, the solution is as simple as the expression for the energy and the calculations can be recycled, saving loads of computer time and attaining the best accuracy possible. With this expression it is possible to compute the entropy fractional emissive power, in particular the Median of the spectral entropy distribution, as well as any other percentile.

The fractional emissive power of isotropic blackbody radiation has been recently determined analytically as ref. 20:

11 $${\Im }_{L}=\frac{15}{{\pi }^{4}}\sigma {T}^{4}\{{x}^{3}L{i}_{1}({e}^{-x})+3{x}^{2}L{i}_{2}({e}^{-x})+6xL{i}_{3}({e}^{-x})+6L{i}_{4}({e}^{-x})\}$$

The polylogarithmic term of Equation 11 when the whole spectrum is studied is $\frac{{\pi }^{4}}{15}$, and therefore the total flux is given by the well known Stefan-Boltzmann’s law *σT* ^4^. Dividing Equation 11 by the total energy flux *σT* ^4^, the normalized fractional emissive power is then determined as:

12 $${\Im }_{L,norm}=\frac{15}{{\pi }^{4}}\{{x}^{3}L{i}_{1}({e}^{-x})+3{x}^{2}L{i}_{2}({e}^{-x})+6xL{i}_{3}({e}^{-x})+6L{i}_{4}({e}^{-x})\}$$

The entropy fractional emissive power of isotropic radiation has not been analysed up to the date. Using a similar procedure, it is determined here as (see Appendix D):

13 $${\Im }_{S}=\frac{15}{{\pi }^{4}}\sigma {T}^{3}\{{x}^{3}L{i}_{1}({e}^{-x})+4{x}^{2}L{i}_{2}({e}^{-x})+8xL{i}_{3}({e}^{-x})+8L{i}_{4}({e}^{-x})\}$$

The total flux is obtained when the whole spectrum is considered. In such situation, the polylogarithmic term is reduced to $\frac{4{\pi }^{4}}{45}$ and the total entropy flux is given by the equivalent Stefan-Boltzmann’s law $\frac{4}{3}\sigma {T}^{3}$. The emissive power normalized to the total entropy flux is therefore given by:

14 $${\Im }_{S,norm}=\frac{45}{4{\pi }^{4}}\{{x}^{3}L{i}_{1}({e}^{-x})+4{x}^{2}L{i}_{2}({e}^{-x})+8xL{i}_{3}({e}^{-x})+8L{i}_{4}({e}^{-x})\}$$

It is noticeable that software such as *Mathematica* gives a more complex solution, which can be proved to be the same when only the real part is considered, differing only in an arbitrary integration constant^23^. The solution provided here is the simplest solution available, and has the advantage that the terms required for its calculation are the same than those required for the energy fractional emissive power, which is translated in much less computational power.

**Figure 2:**
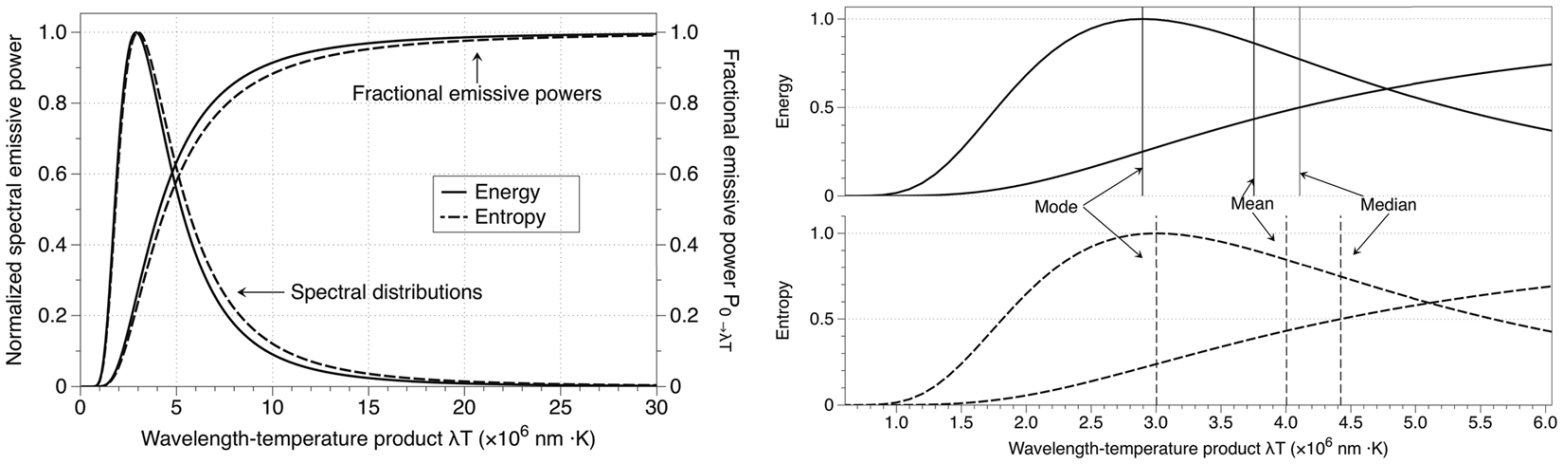
(**a**) (left): Normalized spectral energy and entropy distributions and their corresponding fractional emissive power. (**b**) (right): Zoom including central tendency values: Mode (energy $\simeq$ 2.89777, entropy $\simeq$ 3.00292), Mean (energy $\simeq$ 3.75447, entropy $\simeq$ 4.00477) and Median (energy $\simeq$ 4.10731, entropy $\simeq$ 4.42206).

Figure 2a represents the normalized energy and entropy distributions as well as their corresponding emissive power. Using Equation 14, the Median is determined as the value of *x* which divides the area under the distribution curve into two equal halves:

15 $$\begin{array}{ccc}{\int }_{0}^{{x}_{med}}{S}_{x}dx & = & \frac{45}{4{\pi }^{4}}\{{x}_{med}^{3}L{i}_{1}({e}^{-{x}_{med}})+4{x}_{med}^{2}L{i}_{2}({e}^{-{x}_{med}})+8{x}_{med}L{i}_{3}({e}^{-{x}_{med}})\\  &  & +8L{i}_{4}({e}^{-{x}_{med}})\}=0.5\end{array}$$

The solution of the previous equation is *x*_*med*_ = 3.2536847. Undoing the change of variable $x=\frac{hc}{k\lambda T}$, the value of *λT* = 4.4220637 (×10^6^ nm K) is obtained.

## The Mean and higher moments: statistical characterization of the entropy of radiation

For an absolutely continuous distribution, the Mean is defined as the moment of order one:

16 $${\int }_{0}^{\infty }x\cdot {S}_{x}dx$$

In the case of the entropy of radiation, the integral can be divided as (see Appendix E):

17 $${\int }_{0}^{{\rm{\infty }}}x\cdot {S}_{x}dx=\frac{45}{4{\pi }^{4}}[{\int }_{0}^{{\rm{\infty }}}{x}^{4}\frac{1}{{e}^{x}-1}dx+{\int }_{0}^{{\rm{\infty }}}({x}^{4}+{x}^{3}{\rm{l}}{\rm{o}}{\rm{g}}(\frac{1}{{e}^{x}-1}))dx]$$

The first integral on the right side is the same than in the case of the energy –Bose-Einstein integral– and the second one can be solved in terms of polylogarithms. Solving Equation 17:

18 $${\int }_{0}^{\infty }x\cdot {S}_{x}dx=\frac{45}{4{\pi }^{4}}[6\zeta \mathrm{(5)}+24\zeta \mathrm{(5)}]\simeq 3.59272$$

being *ζ*(*n*) the Riemann zeta function. Undoing the change of variable, the Mean of the entropy of radiation in the *λT* product is $\simeq$4.00477 (×10^6^ nm K). Figure 2b shows a representation of the Mode, Median and Mean for the energy and entropy distributions and their differences.

Besides these three measures of central tendency, the same formalism can be applied to estimate the most important measures of variability. The moments of order two, three and four are determined in Appendix F, and with those calculations the Variance is determined as:

19 $$Var(x)=E[{x}^{2}]-{(E[x])}^{2}=\frac{45}{4{\pi }^{4}}144\zeta (6)-{(\frac{45}{4{\pi }^{4}}30\zeta (5))}^{2}\simeq 4.01172$$

which in the *λT* variable is equal to 3.58649.

The Skewness of the distribution is:

20 $${\gamma }_{1}=E[{(\frac{X-\mu }{\sigma })}^{3}]=\frac{E[{x}^{3}]-3\mu {\sigma }^{2}-{\mu }^{3}}{{\sigma }^{3}}\simeq 1.02192$$

where *σ* is the standard deviation, $\sigma =\sqrt{Var(x)}$. The distribution has positive skew, as inferred from the shape of Fig. 1.

Finally, the Kurtosis is determined as:

21 $$Kurt[X]=\frac{E[{(X-\mu )}^{4}]}{{(E[(X-\mu {)}^{2}])}^{2}}=\frac{E[{x}^{4}]-4E[{x}^{3}]\mu +6E[{x}^{2}]{\mu }^{2}-3{\mu }^{4}}{{\sigma }^{4}}\simeq 4.51404$$

In general, the moment of order *n* of the radiation entropy distribution can be calculated as:

22 $$E[{x}^{n}]=\frac{45}{4{\pi }^{4}}[\frac{4+n}{4+n-1}{\rm{\Gamma }}(4+n)\zeta (4+n)],\,n\ge 1$$

Table 2 summarizes the statistical properties of the entropy and the energy.

**Table 2:** Statistical description of the energy and the entropy of radiation in $x$ and in the *λ*T variable (×10^6^ nm K).

	Energy	Entropy	Energy	Entropy
Mode	*λT* $\simeq$ 2.89777	*λT* $\simeq$ 3.00292	x $\simeq$ 4.96511	x $\simeq$ 4.79127
Mean	*λT* $\simeq$ 3.75447	*λT* $\simeq$ 4.00477	x $\simeq$ 3.83223	x $\simeq$ 3.59272
Median	*λT* $\simeq$ 4.10731	*λT* $\simeq$ 4.42206	x $\simeq$ 3.50302	x $\simeq$ 3.25369
Variance	*λT* $\simeq$ 3.49795	*λT* $\simeq$ 3.58649	x $\simeq$ 4.11326	x $\simeq$ 4.01172
Skewness	*λT* $\simeq$ 14.5853	*λT* $\simeq$ 14.0794	x $\simeq$ 0.98647	x $\simeq$ 1.02192
Kurtosis	*λT* $\simeq$ 3.24557	*λT* $\simeq$ 3.18739	x $\simeq$ 4.43312	x $\simeq$ 4.51404

## Applications

### Exergy fractional emissive power

One of the issues that held back the analysis of the entropy of radiation was the lack of direct applications. This limitation was overcome when the analysis of the maximum efficiency of radiation started and the exergy concept was introduced^7, 24^. The exergy of radiation is a measure of the maximum obtainable work from radiation^25^, i.e., it gives a measure of the useful work, and it involves the determination of the entropy directly.

The thermodynamic approach to radiation was also investigated in the field of radiative transfer. Wildt^21^ proposed the entropy radiative transfer equation, and Liu and Chu^26^ determined the exergy radiative transfer equation. Generally, the entropy of radiation can be determined as the sum of incoherent rays. However, under some circumstances the interaction of polarized waves must be studied using the Stokes vectors and Mueller matrices^27^, and the exergy expression is therefore also different and requires an explicit treatment^28^. In this manuscript, I will refer only to incoherent thermal radiation.

Once the fractional emissive power of the energy and the entropy are known, it is possible to determine the fractional emissive power of the exergy of radiation. The exergy of the whole spectrum for blackbody radiation is well known, but generally solar cells do not cover the whole spectrum. Rather, solar cells are designed to cover only a fraction of it, and the exergy obtainable in that spectral range had to be determined numerically until now.

The spectral distribution of the exergy of radiation is defined as ref. 29:

23 $$E{x}_{\lambda }={L}_{\lambda }(T)-{L}_{\lambda }({T}_{0})-{T}_{0}[{S}_{\lambda }(T)-{S}_{\lambda }({T}_{0})]$$

On the contrary to energy and entropy, exergy is a magnitude which depends on two variables. It depends on the temperature of the emitter, *T*, but also on the temperature of the receiving body, *T*_0_. Naming $x=\frac{hc}{\lambda kT}$ and $y=\frac{hc}{\lambda k{T}_{0}}$, using Equations 11 and 13, and canceling all the possible terms, the simplest analytical expression for the exergy fractional emissive power is:

24 $$\begin{array}{rcl}{\Im }_{E{x}_{0\to {\lambda }_{i}}} & = & \frac{15}{{\pi }^{4}}\sigma \{{T}^{3}[(T-{T}_{0}){x}^{3}L{i}_{1}({e}^{-x})+\mathrm{(3}T-4{T}_{0}){x}^{2}L{i}_{2}({e}^{-x})\\  &  & +\,\mathrm{(6}T-8{T}_{0})xL{i}_{3}({e}^{-x})+(6T-8{T}_{0})L{i}_{4}({e}^{-x})]\\  &  & +{T}_{0}^{4}[{y}^{2}L{i}_{2}({e}^{-y})+2yL{i}_{3}({e}^{-y})+2L{i}_{4}({e}^{-y})]\}\end{array}$$

when *λ*_*i*_ = 0, the values of the energy and the entropy of both bodies are zero; when the whole spectrum is consider, ${\lambda }_{i}=\infty$ → *x* = *y* = 0. In such case, by making use of the relation between the polylogarithms and the Riemann zeta function (see Appendix C), $L{i}_{s}({e}^{0})=L{i}_{s}\mathrm{(1)}=\zeta (s)$, and knowing that $\zeta \mathrm{(4)}=\frac{{\pi }^{4}}{90}$, the exergy of the whole spectrum is reduced to:

25 $$\begin{array}{rcl}{\Im }_{E{x}_{0\to \infty }} & = & \sigma {T}^{4}-\frac{4}{3}\sigma {T}^{3}{T}_{0}+\frac{1}{3}\sigma {T}_{0}^{4}\\  & = & \sigma ({T}^{4}-{T}_{0}^{4})-\frac{4}{3}\sigma {T}_{0}({T}^{3}-{T}_{0}^{3})\end{array}$$

In agreement with previous research^29^. The second law efficiency for the conversion of radiation to work when the whole spectrum is considered is therefore the equation proposed by Petela:

26 $$W=\frac{{{\rm{\Im }}}_{E{x}_{0\to {\rm{\infty }}}}}{{{\rm{\Im }}}_{{L}_{0\to {\rm{\infty }}}}}=\frac{{{\rm{\Im }}}_{E{x}_{0\to {\rm{\infty }}}}}{\sigma {T}^{4}}=1-\frac{4}{3}\frac{{T}_{0}}{T}+\frac{1}{3}{(\frac{{T}_{0}}{T})}^{4}$$

Consequently, using the fractional emissive power of exergy derived in Equation 24, the optimal efficiency or second law efficiency for the conversion of radiation to work in a given spectral region can be defined as:

27 $${W}_{{\lambda }_{1}\to {\lambda }_{2}}=\frac{{\Im }_{E{x}_{{\lambda }_{1}\to {\lambda }_{2}}}}{{\Im }_{{L}_{0\to \infty }}}$$

**Figure 3:**
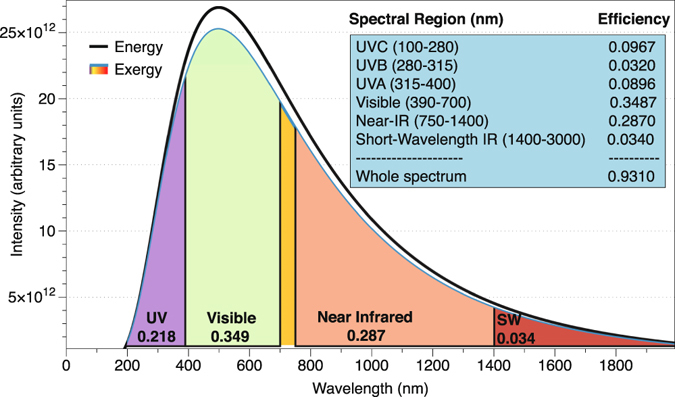
Energy and Exergy of two blackbodies at temperatures *T* = 5800 K and *T*_0_ = 300 K, along with the second law efficiency or exergy efficiency of different spectral regions.

By definition, the exergy obtained by the receiving body is always lower than the energy radiated by the emitting blackbody, as a consequence of the entropy content in radiation. Figure 3 shows a simulation of the energy and the exergy spectra for blackbodies at temperatures *T* = 5800 K and *T*_0_ = 300 K. Equation 26 gives the value for the whole spectrum, whereas the second law efficiency of different spectral regions is analytically calculated using Eq. 27. In the graphic, the value of this efficiency is shown for different regions of the electromagnetic spectrum.

Thus, as a consequence of the entropy content, not all the radiation reaching the Earth’s surface is “useful” to produce work. Therefore, the efficiency of a process involving radiation should be measured against its exergy, not its energy. A particularly interesting process which makes use of radiation is photosynthesis. The photosynthetically active radiation (PAR) region is defined as the spectral range of solar radiation that photosynthetic organisms are able to use in the process of photosynthesis, comprehended in the region from 400 to 700 nanometers. The efficiency of this radiation is usually defined as:

28 $${\eta }_{PAR}(T)=\frac{{\int }_{{\lambda }_{1}}^{{\lambda }_{2}}L(\lambda ,T)d\lambda }{{\int }_{0}^{\infty }L(\lambda ,T)d\lambda }$$

where *L*(*λ*, *T*) stands for the Planck’s function given in Eq. 1. Considering the Sun as a blackbody at 5800 K, the value of this efficiency is *η*_*PAR*_ = 0.368.

However, the organisms which are using solar radiation are also emitting radiation as a consequence of their own temperature. Therefore, the conversion factor of the organism will be different depending on its temperature, and the exergy concept is more suitable than the energy one. By making use of Eq. 27 in the region from *λ*_1_ = 400 nm to *λ*_2_ = 700 nm, the second law PAR conversion factor for a blackbody at *T* = 5800 K and an organism at *T*_0_ = 300 K is calculated as:

29 $${\eta }_{PAR}^{ex}(T)=\frac{{\int }_{{\lambda }_{1}}^{{\lambda }_{2}}Ex(\lambda ,T)d\lambda }{{\int }_{0}^{\infty }L(\lambda ,T)d\lambda }=0.337563$$

which is about an 8.3% lower than the value considered until now. This definition of second law PAR efficiency is more suitable for the analysis of photosynthesis efficiency, since it has in consideration the fact that not all the radiation is useful and takes into account the environmental temperature^30^.

### Earth’s radiation entropy spectrum

As we have seen, the distributions of the energy and the entropy are different and the Mode occurs at different positions. The two magnitudes have different meanings and units, and cannot be compared directly. For such comparison, Fig. 1a showed the normalized spectra of energy and entropy, as well as the ratio entropy to energy (black line). By normalizing to the maximum value of the distribution, dimensionless quantities suitable for comparison are obtained:

30 $${\rm{ratio}}=\frac{S(T)/{S}_{{\rm{\max }}}}{L(T)/{L}_{{\rm{\max }}}}$$

It can be inferred from Fig. 1a that the entropy content in radiation, i.e. the ratio, increases monotonically with wavelength. At certain point, the ratio becomes the unity, and beyond that point, the content of entropy in radiation increases: the entropy content is lower than the unity for high energy photons, and rapidly increases in the infrared.

**Figure 4:**
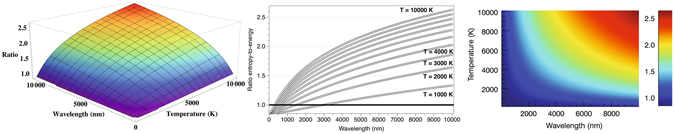
(**a**) (left): 3D plot of the ratio entropy-to-energy as a function of Wavelength and Temperature. (**b**) (center): Projection of the ratio entropy-to-energy in the Wavelength-Ratio space. (**c**) (right): Projection of the ratio entropy-to-energy in the Wavelength-Temperature space.

As the shapes of the distributions are known, an analytical analysis of the ratio distribution can be achieved. Figure 4a shows the surface of the ratio in the Wavelength-Temperature space. One thing that emerges from the graphic is the symmetry between the two variables, which suggests it is possible to reduce the dimension of the analysis, as will be seen later when a new variable *x* = *λT* is introduced. Other conclusion from the graphic is that, for a given wavelength, the ratio increases with temperature, as better seen in Fig. 4b.

Besides the symmetry, it is also possible to see that the iso-entropic surfaces have a clear form, which is better seen looking at the projection represented in Fig. 4c. In this figure, the iso-entropic surfaces are clearly seen as hyperbolic curves, meaning that a determination of the entropy content in radiation can be achieved as a function of the wavelength and the temperature of the blackbody.

Appendix B shows the procedure to obtain the transcendental equation which leads to the determination of the mentioned iso-entropic curves:

31 $${e}^{x}+\frac{{e}^{x}-1}{x}\cdot {\rm{l}}{\rm{o}}{\rm{g}}(\frac{1}{{e}^{x}-1})=n\cdot 1.204196$$

where *x* = *λT* and *n* is the value of the ratio in which one is interested. For example, the curve that divides the space in entropy greater or lower than unity is obtained by doing *n* = 1 in the previous equation and solving it. The solution of such transcendental equation gives a value of *x* = 4.878482, and undoing the change of variable the equation obtained is:

32 $$\lambda T=\frac{hc}{k\cdot 4.8784820}=2.949\times {10}^{6}\,{\rm{nm}}\,{\rm{K}}$$

This function divides the spectra in two parts. The area below the curve represents those values of the wavelength at which the energy is larger than the entropy (deep blue color in Fig. 4c), whereas in the area above the function the entropy is larger than the energy (green and red region in Fig. 4c). The curve represents the wavelength-temperature combinations at which the ratio is exactly one.

This kind of curve can be easily obtained for different values of the ratio. In Table 3, I summarize the coefficient for a variety of values of the ratio. If more accuracy is required, the coefficient can be obtained by solving Equation 31 directly.

**Table 3:** Coefficients for the different values of the ratio entropy to energy law obtained by solving Eq. 31: *λT* = coefficient (×10^6^ nm K).

	0.0	0.1	0.2	0.3	0.4	0.5	0.6	0.7	0.8	0.9
0	—	—	—	—	—	—	—	—	—	1.205
1	2.949	4.792	6.838	9.162	11.823	14.876	18.378	22.395	26.993	32.252
2	38.258	45.109	52.914	61.797	71.898	83.374	96.402	111.1823	127.941	146.930
3	168.438	192.788	220.344	251.518	286.773	326.634	371.670	422.607	480.135	545.122

Integers are in the left column and decimals in the upper row. For example, the coefficient for ratio equal to 2.4 is 71.898 × 10^6^ nm K.

One interesting thing of the analysis of the entropy content in radiation is the fact that there is a minimum value of the ratio. In this analysis in the wavelength representation, the transcendental equation does not have solution for a ratio below 0.86. In the numerical analysis, using double precision variables in fortran, the obtained value of the minimum was 0.830429, which corresponds to the blue region in the bottom left corner in Fig. 4c. When working with classical equations such as 1–4, one should keep in mind the range of validity of those predictions (thermal radiation), since extreme processes such as X-rays are not the scope of the theory.

In this paper, I have made use of blackbody radiation, which is defined as radiation with the maximum possible amount of entropy for a given energy. By making use of Eq. 31 and Table 3, it is possible to compare radiation from different sources to blackbody radiation, i.e., they provide a general way to measure deviations of its entropy content from blackbody radiation for the same amount of energy. Although blackbody radiation might be seen as a theoretical ideal, this sort of spectrum is used in a variety of applications in engineering, astrophysics and climate sciences. In particular, the solar and the Earth’s spectra can be approximated by this ideal source of radiation for certain applications.

**Figure 5:**
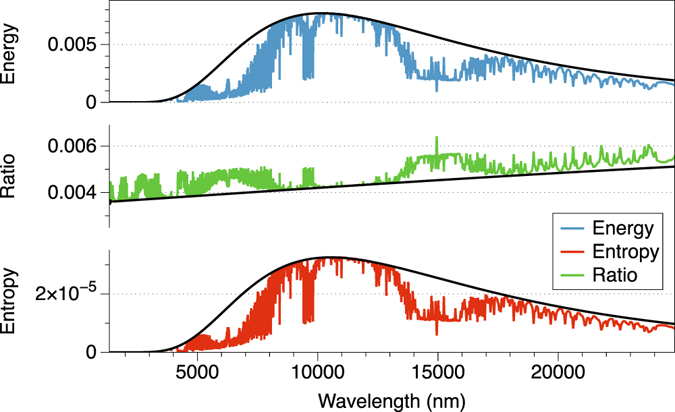
Energy (W/m^2^/nm), entropy (W/m^2^/nm/K) and ratio (*K* ^−1^) of Earth’s upwelling radiation. The black solid line represents a blackbody at temperature *T* = 285 K.

In Fig. 5, I show the entropy (red) and the energy (blue) spectra of the radiation emitted by the Earth. I evaluate the spectral distribution of the energy and the entropy of radiation solving the radiative transfer equation under clear sky conditions to calculate the spectral intensity, and determining the associated entropy afterwards using Eq. 2. I have used FUTBOLIN (Full Transfer By Optimized LINe-by-line)^31^, a validated line by line radiative transfer code, and the standard US atmosphere to generate a simulation of Earth’s current spectrum. I “idealized” its emission as a blackbody at temperature *T* = 285 K (black line in Fig. 5). The simulation does not include scattering or splitting entropies, and the deviations from the blackbody behaviour are due only to emission/absorption processes. It has the intention to show an application, not to provide an in-deep analysis, which will be carried out in future research.

As it can be seen, the ratio of entropy to energy (green line) at certain wavelengths contains more entropy than the expected for a blackbody (black line). This entropy is not a consequence of blackbody emission, and is generated by the irreversible processes that take place in the atmosphere. By making the difference at individual wavelengths between the magnitude of its ratio and the expected for a blackbody, the entropy production in the atmosphere by absorption/emission processes is characterized as the strength of each line. Different chemical species in the atmosphere will produce more or less radiation entropy, and in this way we can actually measure their contribution.

Figure 5 is included here as an example of the uses of radiation entropy, since the spectral behaviour of the radiation entropy on Earth has not been an intense field of study. While some research has been done to analyze the vertical distribution of radiation entropy^22, 32^, not much attention has been paid to its spectral distribution, with the exception of ref. 33. Further research is needed in this field, and this paper provides a methodology to characterize the radiation entropy production in the atmosphere, an important topic in climate sciences.

If the spectral behaviour of the Earth is measured at different epochs, it will be possible to compare their entropy production and actually *measure* the climatic changes in the atmosphere. This approach provides a metric to study climate change directly from a thermodynamical perspective, instead of analyse its effects such as the increase of temperature or the ice layer depletion in the poles. It is beyond the scope of this paper to carry out such analysis, which will be the topic of a separated contribution.

### Entropy, eyesight and solar Wien peaks

Although the entropy concept was originated in physics, there are many other fields which employ this magnitude. In particular, entropy is the cornerstone of information theory^13^, understanding it in relation with the information obtainable from a system^34^. Information theory is currently applied successfully to computer vision problems, and in a previous issue I showed that it can also be applied to human vision^14^. This application of radiation entropy to human vision was made by determining the wavelength of maximum entropy intensity, proving that entropy plays a major role as driving force in the biological evolution of human eyesight.

However, as we have seen, the maximum of the entropy depends on the selected spectral variable. In order to know if entropy is of importance in the evolution of human eye, it is necessary to analyze the integral of the function, not its maximum alone. Even though the spectral distribution of entropy depends on the variable, its integral does not, being more physically meaningful than the density itself.

Here I analyze which would be the best balance between the wavelength of peak sensitivity and the range of wavelengths that human eye is capable of responding, approximately Δ*λ* = 300 nm from 400 to 700 nm, following the reasoning in ref. 15. The question I am trying to answer is: which would be the optimum peak sensitivity (*λ*_*p*_) knowing that our retina can only catch photons in a determined region (Δ*λ*)?

**Figure 6:**
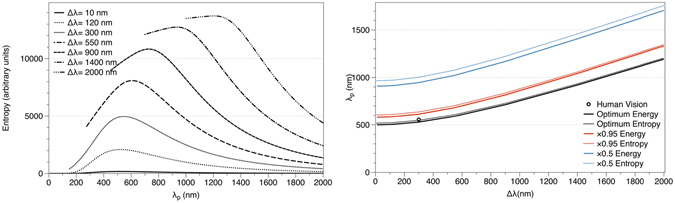
(**a**) (left): Entropy of solar radiation in the range *λ*_*p*_ ± Δ*λ* for different values of Δ*λ*. (**b**) (right): Contours of equal solar intensity (continuous) and entropy (dotted).

Following Overduin’s reasoning^15^, we can investigate which would be the optimal case for human vision to adapt to for a variety of wavelength intervals. Using Equation 13, I determine the entropy of solar radiation emitted in the spectral range *λ*_*p*_ ± Δ*λ*/2 for values of Δ*λ* = 10, 120, 300, 500, 900, 1400 and 2000 nm, plotted in Fig. 6a.

Figure 6 should be understood as follows. Figure 6a gives the entropy flux if our retinas had an amplitude of Δ*λ* = 10 nm, or 120 nm, or 300 nm …. The wavelength of “peak” sensitivity depends on the spectral range, and its maximum is plotted in Fig. 6b. The black line in Fig. 6b shows the position (peak sensitivity) of the maxima depending on the spectral range. The red line shows the peak sensitivity if our retinas only catch 95% of the intensity, and the blue line does the same for 0.5 times the intensity.

In Fig. 6b the actual location of the human vision is represented by a circle, the optimal energy by a black continuous line and the optimal entropy by a black dotted line; the solar spectrum has been modelled as a blackbody at 5800 K, to be consistent with the previous research^14^. In such case, as the range of wavelengths of human eyesight is about Δ*λ* = 300 nm, the peak sensitivity should be approximately at 547 nm. As seen in the figure, human vision matches the line of optimum entropy better, suggesting that, indeed, entropy plays a major role as driving force in the evolution of human eye, and not only the energy as previously thought.

The other lines in Fig. 6b represent those combinations of *λ*_*p*_ and Δ_*λ*_ where the intensity and entropy are equal to 0.95 or 0.5 times the maximal. Similarly, continuous lines correspond to energy and dotted lines to entropy. Human eyesight is not adapted to those lines, but instead is adapted to the maximum entropy intensity. This analysis of the entropy adds value to the idea that human eyesight evolved with a follow-the-information rule looking for the maximum entropy, not only for the maximum energy, which opens new ways to understand human perception and inference.

## Conclusions

In this paper, I showed that the entropy of radiation distribution has its maximum in a different location than the energy distribution. The Mode of the distribution can be determined by a Wien’s like law and I provided the coefficients for a variety of dispersion rules, including wavelength and frequency.

Usually, the fractional emissive power is determined numerically, but analytical solutions are always preferred. In this paper, I did the integration of the spectral entropy in a given region using polylogarithmic functions, obtaining the classical thermodynamic relations when the whole spectrum is considered. The Median and the Mode are obtained and compared to the energy values, and variability measures are provided.

By making use of the derived expressions, I calculated the radiation exergy spectral power and define the second law efficiency in a region. This definition is of importance in any process involving radiation, and in particular in photosynthesis. The second law efficiency for the photosynthetically active radiation (PAR) region is defined and determined, showing that it is lower than previously considered, as a consequence of the temperature of the organism.

Blackbody radiation is defined as that containing the largest amount of entropy for a given energy. By comparing the Earth’s upwelling radiation spectrum with respect to a blackbody at 285 K, I showed how to obtain the magnitude of the entropy production in the atmosphere at different wavelengths, which opens new lines to investigate atmospheric processes and climate.

Entropy concept was originated in physics but its application has spread to many different fields, in particular information theory. From that point of view, it has been recently proposed that entropy is a driving force in the evolution of human eyesight. With the application of the expressions obtained in this paper, I proved how the influence of the entropy is independent of the spectral variable, showing a new approach to analyze radiation processes and its effect on biological systems and their evolution.

The equations obtained in this paper are fully developed in the appendixes, which hopefully will encourage researchers to explore this field and its interdisciplinary connections.

## Electronic supplementary material

Supplementary Information: Appendixes
