# Supplementary material for: Entropy of radiation: the unseen side of light
Source: Sci Rep. 2017 May 10;7:1642. doi: 10.1038/s41598-017-01622-6 (PMC5432030; doi:10.1038/s41598-017-01622-6)
Supplement: Supplementary file 1 — Supplementary Information: Appendixes [file 41598_2017_1622_MOESM1_ESM.pdf]

# Entropy of radiation: the unseen side of light

**Alfonso Delgado-Bonal**[1]

[1] University of Salamanca
Institute of Fundamental Physics and Mathematics
Pza. de la Merced S/N, 37008, Salamanca, Spain

alfonso_delgado@usal.es

April 24, 2017

## Supplementary Information: Appendixes

## Contents

**Historical development of radiation entropy** **2**

**A Transcendental equation for the Mode** **4**

**B Ratio of normalized entropy to energy** **7**

**C Polylogarithms** **9**

**D Integral of the spectral entropy of radiation** **10**

**E Mean of the energy and entropy of radiation** **12**

**F Higher moments: Variance, Skewness and Kurtosis** **14**

## Historical development of radiation entropy

The standard story of the development of the radiation law and the beginning of the quantum theory is usually told in terms of energy, when the reality is that the thermodynamical entropy played the main role in the analysis of radiation. The unfortunate fate of the entropy was to be buried under an unrealistic history about the failure of classic physics, profound disagreements between theory and experiments and the so called ultraviolet catastrophe; that the energy quanta were then introduced –almost magically– by a prodigious Max Planck, and quantum mechanics became generally accepted immediately.

The truth is that Wien's law was believed to be correct at the time; only a slight disagreement was found for long wave radiation, and the Rayleigh-Jeans equation and the "Ultraviolet catastrophe" (named by Ehrenfest in 1911) come across years after Planck's proposition of his law. The quantum theory and the radiation law were the result of the mastering of thermodynamics by Boltzmann, Wien, Planck, Einstein and their contemporaries.

According to the electromagnetic theory, the energy distribution law was determined as soon as the entropy $S$ of a linear resonator which interacts with the radiation were known as function of the vibrational energy $U$. Wien was aware of the importance of the entropy [1], and so was Planck.

In October 19th 1900, Max Planck presented in front of the German Physical Society the law which determines the spectral distribution of blackbody radiation, obtained as an interpolation of Wien's law [2]. In December 14th 1900 he presented the statistical justification of the formula by introducing discrete energy elements, making a novel use of the Boltzmann's statistical definition of the entropy [3]. The equation leaded to the development of the quantum theory and it has been vastly studied in the context of radiative transfer.

At the very end of the XIX century, Wien's law [4] was proven valid for short wavelengths but not completely accurate for the whole spectra and, on the other hand, Rayleigh had proposed a formula valid for long wave radiation [5]. From Rayleigh's formula, the relation between entropy and energy was of the kind:

$$\frac{\partial^2 S}{\partial^2 U} = \frac{\text{const.}}{U} \tag{1}$$

The expression on the right-hand side of this functional equation is the change in entropy since $n$ identical processes occur independently, the entropy changes of which must simply add up [2].

On the other hand, from Wien's distribution law the relation would be something of the sort:

$$\frac{\partial^2 S}{\partial^2 U} = \frac{\text{const.}}{U^2} \tag{2}$$

Analyzing a variety of completely arbitrary expressions, Planck proposed the simplest equation (besides the Wien's one) which yield $S$ as a logarithmic function of $U$, and

coincided with the Wien's law for small values of $U$. The logarithmic relation was a constraint from the probability theory of Mr. Boltzmann, whose works were known by Planck and were the base upon which the theory was developed afterwards. Without further justification, Planck included a new term as a series expansion, proportional to $U^2$:

$$\frac{\partial^2 S}{\partial^2 U} = \frac{\text{const.}}{U(\beta + U)} \tag{3}$$

Using this expression and the relation $\partial S/\partial U = 1/T$, one gets a radiation formula with two constants:

$$U = \frac{C_1 \lambda^{-5}}{e^{C_2/\lambda T} - 1} \tag{4}$$

By making use of the available data to fit the constants, the equation resulted in the nowadays named Planck's law.

The formula was, indeed, exact, but it was obtained without any underlying theory, so Planck devoted himself to the task of constructing a radiation theory on the base of Boltzmann's statistical mechanics and the logarithmic expression of the entropy:

$$S = k \log W \tag{5}$$

where $S$ is the thermodynamic entropy, $W$ is the number of possible microstates, and $k$ is Boltzmann's constant (introduced later by Planck). In a system composed by incoherent radiation beams [6][7], the total entropy can be expressed as the sum $S = S_1 + S_2$ which implies that:

$$W = W_1 \cdot W_2 \tag{6}$$

where $W$ is the number of ways in which one can distribute $P$ energy elements over $N$ hypothetical resonators. Using combinatory analysis, Planck obtained the expression for the microstates [3], and using the Boltzmann's entropy expression, he obtained the entropy distribution [8]:

$$S = k\left[(N+P)\ln(N+P) - N\ln N - P\ln P\right] \tag{7}$$

As the aim of Planck was to obtain the energy distribution, he made the marvelous hypothesis of discrete energy, $\epsilon = h\nu$, motivated by Boltzmann's works, obtaining the expression:

$$S = k\left[\left(1 + \frac{U}{h\nu}\right)\ln\left(1 + \frac{U}{h\nu}\right) - \frac{U}{h\nu}\ln\frac{U}{h\nu}\right] \tag{8}$$

At this point, differentiating with respect to $U$ and using the relation $\partial S/\partial U = 1/T$, Planck obtained:

$$\frac{1}{T} = \frac{k}{h\nu}\ln\left(1 + \frac{h\nu}{U}\right) \tag{9}$$

which directly gave him the expression for the energy distribution law that he was looking for:

$$U = \frac{h\nu}{exp(h\nu/kT) - 1} \tag{10}$$

With this expression, going back to the entropy, the spectral entropy of radiation is [6]:

$$S_\nu = \frac{k\nu^2}{c^2}\left\{\left(1+\frac{1}{e^{\frac{h\nu}{kT}}-1}\right)\log\left(1+\frac{1}{e^{\frac{h\nu}{kT}}-1}\right)-\frac{1}{e^{\frac{h\nu}{kT}}-1}\log\left(\frac{1}{e^{\frac{h\nu}{kT}}-1}\right)\right\} \tag{11}$$

This expression was obtained later by many other ways [7][9][10] but, interestingly, the use of the entropy in the analysis of radiation is mainly forgotten nowadays. However, the situation was quite different in the past, and several Nobel laureates worked and published research related directly to the topic, such as Wien [1], Planck [6], Einstein [7], von Laue [11], Lorentz [12] or Kastler [13] among others.

# A Transcendental equation for the Mode

The Planck's law in frequency is:

$$L_\nu = \frac{2h\nu^3}{c^2}\frac{1}{e^{\frac{h\nu}{kT}}-1} \tag{12}$$

The expression for the entropy is more complex, and corresponds to the entropy of bosons. Prior to the derivative, it is a good idea to manipulate the expression arithmetically, rewriting it as:

$$\begin{aligned}
S_\nu &= \frac{2k\nu^2}{c^2}\left\{\left(1+\frac{1}{e^{\frac{h\nu}{kT}}-1}\right)\log\left(1+\frac{1}{e^{\frac{h\nu}{kT}}-1}\right)-\frac{1}{e^{\frac{h\nu}{kT}}-1}\log\left(\frac{1}{e^{\frac{h\nu}{kT}}-1}\right)\right\}\\
&= \frac{2k\nu^2}{c^2}\cdot\frac{1}{e^{\frac{h\nu}{kT}}-1}\left\{e^{\frac{h\nu}{kT}}\log\left(\frac{e^{\frac{h\nu}{kT}}}{e^{\frac{h\nu}{kT}}-1}\right)-\log\left(\frac{1}{e^{\frac{h\nu}{kT}}-1}\right)\right\}\\
&= \frac{2k\nu^2}{c^2}\cdot\frac{1}{e^{\frac{h\nu}{kT}}-1}\left\{e^{\frac{h\nu}{kT}}\cdot\log e^{\frac{h\nu}{kT}}+(e^{\frac{h\nu}{kT}}-1)\cdot\log\left(\frac{1}{e^{\frac{h\nu}{kT}}-1}\right)\right\}\\
&= \frac{2k\nu^2}{c^2}\cdot\frac{1}{e^{\frac{h\nu}{kT}}-1}\left\{e^{\frac{h\nu}{kT}}\frac{h\nu}{kT}+(e^{\frac{h\nu}{kT}}-1)\cdot\log\left(\frac{1}{e^{\frac{h\nu}{kT}}-1}\right)\right\}\\
&= \frac{2k\nu^2}{c^2}\cdot\frac{h\nu}{kT}\frac{e^{\frac{h\nu}{kT}}}{e^{\frac{h\nu}{kT}}-1}+\frac{2h\nu^2}{c^2}\log\left(\frac{1}{e^{\frac{h\nu}{kT}}-1}\right)
\end{aligned}$$

$$
\begin{aligned}
&= \frac{2h\nu^3}{c^2T} + \frac{2h\nu^3}{c^2T}\frac{1}{e^{\frac{h\nu}{kT}}-1} + \frac{2h\nu^2}{c^2}\log\left(\frac{1}{e^{\frac{h\nu}{kT}}-1}\right) \\
&= \frac{2h\nu^3}{c^2T} + \frac{1}{T}\cdot L_\nu + \frac{2k\nu^2}{c^2}\cdot\log\left(\frac{1}{e^{\frac{h\nu}{kT}}-1}\right)
\end{aligned} \tag{13}
$$

In this way, the second term is the same than in the case of the energy, and the calculations can be recycled. Doing $dS/d\nu = 0$ and removing a factor of 2:

$$
\begin{aligned}
\frac{dS}{d\nu} = \frac{3h\nu^2}{c^2T} &+ \frac{3h\nu^2}{c^2T\left(e^{\frac{h\nu}{kT}}-1\right)} - \frac{h^2\nu^3}{kT^2c^2}\frac{e^{\frac{h\nu}{kT}}}{\left(e^{\frac{h\nu}{kT}}-1\right)^2} \\
&+ \frac{2k\nu}{c}\cdot\log\left(\frac{1}{e^{\frac{h\nu}{kT}}-1}\right) - \frac{h\nu^2}{c^2T}\frac{e^{\frac{h\nu}{kT}}}{\left(e^{\frac{h\nu}{kT}}-1\right)} = 0
\end{aligned} \tag{14}
$$

As in the case of the energy, one can appreciate that frequency corresponds to the dispersion coefficient $m = 3$, wich will be seen later. Continuing with the derivative, looking for common denominator with the term $\frac{1}{c^2T^2\left(e^{\frac{h\nu}{kT}}-1\right)^2}$ and manipulating the latest expression, we have:

$$
\begin{aligned}
\frac{dS}{d\nu} = &\frac{3h\nu^2T\left(e^{\frac{h\nu}{kT}}-1\right)^2}{c^2T^2\left(e^{\frac{h\nu}{kT}}-1\right)^2} + \frac{3h\nu^2T\left(e^{\frac{h\nu}{kT}}-1\right)}{c^2T^2\left(e^{\frac{h\nu}{kT}}-1\right)^2} - \frac{h^2\nu^3}{kT^2c^2}\frac{e^{\frac{h\nu}{kT}}}{\left(e^{\frac{h\nu}{kT}}-1\right)^2} \\
&+ \frac{2k\nu T^2\left(e^{\frac{h\nu}{kT}}-1\right)^2}{c^2T^2\left(e^{\frac{h\nu}{kT}}-1\right)^2}\cdot\log\left(\frac{1}{e^{\frac{h\nu}{kT}}-1}\right) - \frac{h\nu^2T}{c^2T^2}\frac{e^{\frac{h\nu}{kT}}\left(e^{\frac{h\nu}{kT}}-1\right)}{\left(e^{\frac{h\nu}{kT}}-1\right)^2} = 0
\end{aligned} \tag{15}
$$

From which the numerator must be zero in order to be $dS/d\nu = 0$:

$$
\begin{aligned}
\frac{dS}{d\nu} = &3h\nu^2T\left(e^{\frac{h\nu}{kT}}-1\right)^2 + 3h\nu^2T\left(e^{\frac{h\nu}{kT}}-1\right) - \frac{h^2\nu^3}{k}e^{\frac{h\nu}{kT}} \\
&+ 2k\nu T^2\left(e^{\frac{h\nu}{kT}}-1\right)^2\cdot\log\left(\frac{1}{e^{\frac{h\nu}{kT}}-1}\right) - h\nu^2Te^{\frac{h\nu}{kT}}\left(e^{\frac{h\nu}{kT}}-1\right) = 0
\end{aligned} \tag{16}
$$

Doing the change of variable $x = \frac{h\nu}{kT} \rightarrow \nu = \frac{xkT}{h}$, we have:

$$
\begin{aligned}
\frac{dS}{d\nu} = &3\frac{T^3k^2}{h}x^2\left(e^x-1\right)^2 + 3\frac{T^3k^2}{h}x^2\left(e^x-1\right) - \frac{T^3k^2}{h}x^3e^x \\
&+ 2\frac{T^3k^2}{h}x\left(e^x-1\right)^2\cdot\log\left(\frac{1}{e^x-1}\right) - \frac{T^3k^2}{h}x^2e^x\left(e^x-1\right) = 0
\end{aligned} \tag{17}
$$

which getting ride of the factor $\frac{T^3k^2}{h}\cdot x$ gives the transcendental equation showed in Eq. 6 in the main text. If the same procedure is followed for the wavelength representation, the

result is Eq. 8 in the main text. In the case of choosing the wavelength representation, the "arithmetically transformed" expression for the entropy is shown in the next appendix.

Figure 1 shows the solution of the equation for different dispersion rules. The physical meaning of each dispersion rule is explained in the caption of the figure, and the numerical solution of the equation is shown for each case in Table 1 in the main text.

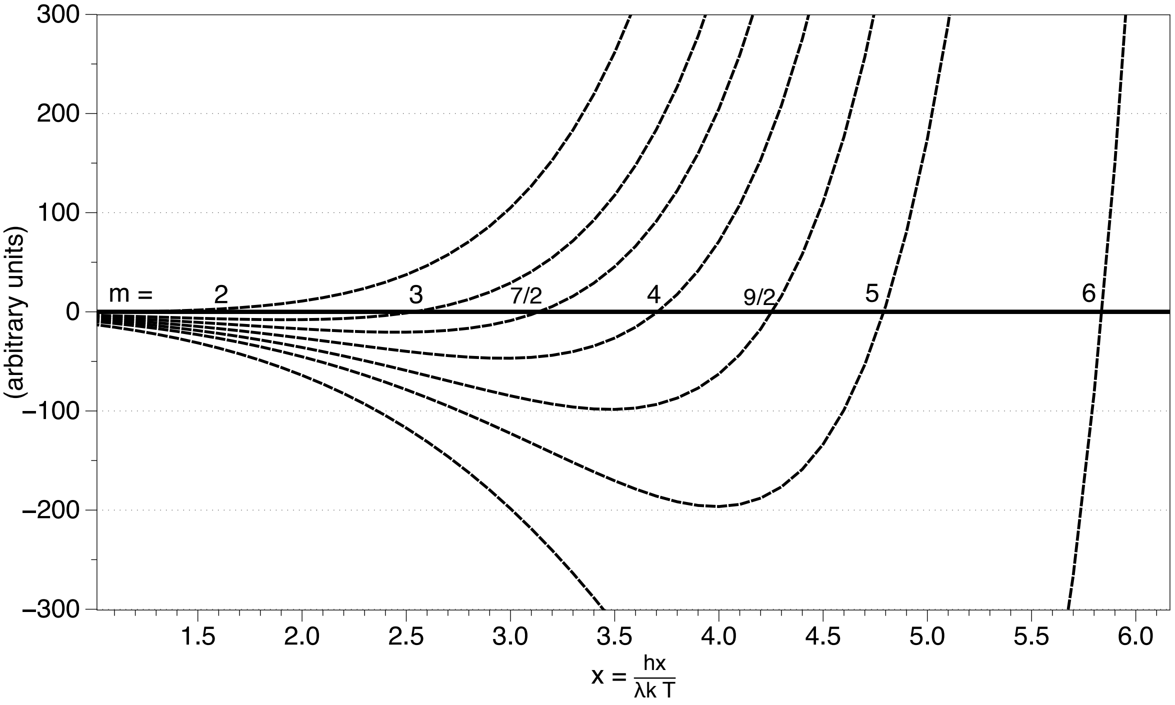


Figure 1: Solution to the transcendental equation as a function of the dispersion coefficient. **m = 2:** $\nu^2$ (frequency-squared); **m = 3:** $\nu$ (linear frequency); **m = 7/2:** $\sqrt{\nu}$ (square root frequency); **m = 4:** $\log \nu$ (logarithmic frequency); **m = 4:** $\log \lambda$ (logarithmic wavelength); **m = 9/2:** $\sqrt{\lambda}$ (square root wavelength); **m = 5:** $\lambda$ (linear wavelength); **m = 6:** $\lambda^2$ (wavelength-squared).

As we have seen in this paper, both energy and entropy follow a Wien's displacement law for the determination of their maxima, but the energy distribution has a particularity which has not been found on the entropy yet.

In this paper, the general transcendental equation for the Mode of the entropy is solved numerically in order to obtain the associated Wien's law. In the case of the energy, the classical procedure to obtain the Wien's law was also the numerical solution of the transcendental equation:

$$(x - m)e^x = -m \tag{18}$$

However, more recent research have shown that it can be solved in terms of the Lambert W function [14]:

$$b_{energy} = \frac{hc/k}{m + W_0(-me^{-m})} \tag{19}$$

The details of this function are perfectly described in [15], and provides an elegant analytical solution to the problem. The general equation obtained in this paper resembles the one obtained for the energy, but its solution in terms of Lambert W function is not straightforward and it is beyond the scope of this paper. Perhaps a skilled reader can solve it, which will provide an analytical beauty to the problem treated in this section.

# B Ratio of normalized entropy to energy

The entropy of radiation distribution in the wavelength representation, as in the previous appendix, can be rewritten as:

$$\begin{aligned}
S_\lambda &= \frac{2kc}{\lambda^4}\frac{1}{e^{hc/\lambda kT}-1}\left\{e^{hc/\lambda kT}\log\left(\frac{e^{hc/\lambda kT}}{e^{hc/\lambda kT}-1}\right) - \log\left(\frac{1}{e^{hc/\lambda kT}-1}\right)\right\} \\
&= \frac{2kc}{\lambda^4}\frac{1}{e^{hc/\lambda kT}-1}\left\{e^{hc/\lambda kT}\log\left(e^{hc/\lambda kT}\right) + (e^{hc/\lambda kT}-1)\log\left(\frac{1}{e^{hc/\lambda kT}-1}\right)\right\} \\
&= \frac{2kc}{\lambda^4}\frac{1}{e^{hc/\lambda kT}-1}\left\{e^{hc/\lambda kT}\frac{hc}{\lambda kT} + (e^{hc/\lambda kT}-1)\log\left(\frac{1}{e^{hc/\lambda kT}-1}\right)\right\} \\
&= \frac{2kc}{\lambda^4}\frac{e^{hc/\lambda kT}}{e^{hc/\lambda kT}-1}\frac{hc}{\lambda kT} + \frac{2kc}{\lambda^4}\log\left(\frac{1}{e^{hc/\lambda kT}-1}\right) \\
&= \frac{2kc}{\lambda^4}\frac{hc}{\lambda kT} + \frac{2kc}{\lambda^4}\frac{1}{e^{hc/\lambda kT}-1}\frac{hc}{\lambda kT} + \frac{2kc}{\lambda^4}\log\left(\frac{1}{e^{hc/\lambda kT}-1}\right)
\end{aligned} \tag{20}$$

Which reduces to:

$$S_\lambda = \frac{2hc^2}{T\lambda^5} + \frac{1}{T}L_\lambda + \frac{2kc}{\lambda^4}\cdot\log\left(\frac{1}{e^{\frac{hc}{\lambda kT}}-1}\right) \tag{21}$$

The value of the ratio of normalized entropy to energy, $n$, is determined by the equation:

$$\frac{S_\lambda/S_{\lambda,max}}{L_\lambda/L_{\lambda,max}} = n \tag{22}$$

Where $S_{\lambda,max}$ and $L_{\lambda,max}$ are the maxima of the entropy and the energy respectively. The relation can be rewritten as:

$$S_\lambda \cdot L_{\lambda,max} = n \cdot S_{\lambda,max} \cdot L_\lambda \tag{23}$$

$S_{\lambda,max}$ and $L_{\lambda,max}$ are determined by their corresponding Wien's laws, so we can use the relations $\lambda_{\text{max,energy}} \cdot T = b_{\text{energy}}$ and $\lambda_{\text{max,entropy}} \cdot T = b_{\text{entropy}}$, expressing the functions at their maxima as:

$$S_{\lambda,\max} = \frac{2hc^2 \cdot T^5}{T \cdot b_{\text{entropy}}^5} + \frac{1}{T}\frac{2hc^2}{b_{\text{entropy}}^5}\frac{T^5}{e^{\frac{hc}{kb_{\text{entropy}}}} - 1} + \frac{2kc}{b_{\text{entropy}}^4} \cdot T^4 \cdot \log\left(\frac{1}{e^{\frac{hc}{kb_{\text{entropy}}}} - 1}\right) \tag{24}$$

$$L_{\lambda,\max} = \frac{2hc^2}{b_{\text{energy}}^5}\frac{T^5}{e^{\frac{hc}{kb_{\text{energy}}}} - 1} \tag{25}$$

For simplicity, calling $c_1 = 2hc^2$, $c_2 = hc/k$ and $c_3 = 2kc$, the relation described in Equation 23 leads to the equation:

$$\begin{gathered}\frac{c_1}{T \cdot \lambda^5} \cdot \frac{c_1}{b_{\text{energy}}^5}\frac{T^5}{\left(e^{c_2/b_{\text{energy}}} - 1\right)} + \frac{1}{T}\frac{c_1}{\lambda^5}\frac{1}{\left(e^{c_2/\lambda T} - 1\right)}\frac{c_1}{b_{\text{energy}}^5}\frac{T^5}{\left(e^{c_2/b_{\text{energy}}} - 1\right)} \\ + \frac{c_3}{\lambda^4} \cdot \log\left(\frac{1}{e^{c_2/\lambda T} - 1}\right) \cdot \frac{c_1}{b_{\text{energy}}^5}\frac{T^5}{\left(e^{c_2/b_{\text{energy}}} - 1\right)} = \\ n \cdot \left\{\frac{c_1}{T}\frac{T^5}{b_{\text{entropy}}^5} \cdot \frac{c_1}{\lambda^5}\frac{1}{\left(e^{c_2/\lambda T} - 1\right)} + \frac{1}{T}\frac{c_1}{b_{\text{entropy}}^5}\frac{T^5}{\left(e^{c_2/b_{\text{entropy}}} - 1\right)}\frac{c_1}{\lambda^5}\frac{1}{\left(e^{c_2/\lambda T} - 1\right)}\right. \\ \left. + \frac{c_3}{b_{\text{entropy}}^4} \cdot T^4 \cdot \log\left(\frac{1}{e^{c_2/b_{\text{entropy}}} - 1}\right) \cdot \frac{c_1}{\lambda^5}\frac{1}{\left(e^{c_2/\lambda T} - 1\right)}\right\}\end{gathered} \tag{26}$$

Removing $\frac{T^4}{\lambda^5}$, naming $x = \frac{c_2}{\lambda T}$ and multiplying both sides by $(e^x - 1)$, the equation is reduced to:

$$\begin{gathered}e^x + \frac{e^x - 1}{x} \cdot \log\left(\frac{1}{e^x - 1}\right) = n \cdot \left\{\left(\frac{b_{\text{energy}}}{b_{\text{entropy}}}\right)^5 \cdot \left(e^{\frac{hc}{kb_{\text{energy}}}} - 1\right) \cdot \right. \\ \left.\left\{1 + \frac{1}{e^{\frac{hc}{kb_{\text{entropy}}}} - 1} + \frac{kb_{\text{entropy}}}{hc} \cdot \log\left(\frac{1}{e^{\frac{hc}{kb_{\text{entropy}}}} - 1}\right)\right\}\right\}\end{gathered} \tag{27}$$

The right side of the equation depends only on the value of the ratio, since the term inside the curl is a constant of an approximate value of 1.204196. Using this value, the equation is approximated to:

$$e^x + \frac{e^x - 1}{x} \cdot \log\left(\frac{1}{e^x - 1}\right) = n \cdot 1.204196 \tag{28}$$

The solution for different values of the ratio is shown in Table 3 of the main text. For example, for a value or ratio equal to unity, the numerical solution of the transcendental equation is $x = 4.878482$. Undoing the change of variable, we have:

$$\lambda_{n=1}T = \frac{hc}{k \cdot 4.8784820} = 2.94923 \cdot 10^{-3}\text{m K} \tag{29}$$

# C Polylogarithms

Detailed information of polylogarithms –an old function known since 250 years ago [16]– can be found for example in [17]. In this appendix the most important properties which are of interest for this work are reviewed.

The polylogarithm is a special function $Li_s(z)$ of order $s$ and argument $z$. For special values of $s$ the polylogarithm is reduced to an elementary function (such as the natural logarithm). Polylogarithms are defined as the infinite sum for arbitrary complex order $s$ and for all complex arguments $z$ [18]:

$$Li_s(z) = \sum_{k=1}^{\infty} \frac{z^k}{k^s} = z + \frac{z^2}{2^s} + \frac{z^3}{3^s} + \dots \tag{30}$$

or as the repeated integral of itself:

$$Li_{s+1}(z) = \int_0^z \frac{Li_s(t)}{t} dt \tag{31}$$

The derivatives follow from the defining power series in Equation 30, and particularly for exponential functions:

$$\begin{aligned} z\frac{\partial Li_s(z)}{\partial z} &= Li_{s-1}(z) \\ \frac{\partial Li_s(e^x)}{\partial x} &= Li_{s-1}(e^x) \\ \frac{\partial Li_s(e^{-x})}{\partial x} &= -Li_{s-1}(e^{-x}) \end{aligned} \tag{32}$$

In the special case $s = 1$, the polylogarithm is reduced to the ordinary natural logarithm:

$$Li_1(z) = -\log(1-z) \tag{33}$$

for $s$ = 0, -1, -2, the polylogarithm is:

$$Li_0(z) = \frac{z}{1-z}; \quad Li_{-1}(z) = \frac{z}{(1-z)^2}; \quad Li_{-2}(z) = \frac{z(1+z)}{(1-z)^3} \tag{34}$$

Some properties of the integral of exponential functions are listed here. Following Equation 31, we have:

$$\int_x^{\infty} Li_s(e^{-t})dt = Li_{s+1}(e^{-x}) \tag{35}$$

and the indefinite integral with an arbitrary constant:

$$\int Li_s(e^{-x})dx = -Li_{s+1}(e^{-x}) \tag{36}$$

Particularly interesting are the integrals of the form [19]:

$$\int x^k Li_0(e^{-x})dx = -\sum_{n=0}^{k} x^{k-n} Li_{n+1}(e^{-x}) \frac{\Gamma(k+1)}{\Gamma(k+1-n)} \tag{37}$$

where $k$ is a non-negative integer and $\Gamma(x)$ is the Euler gamma function.
For $z = 1$ the polylogarithm reduces to the Riemann zeta function:

$$Li_s(1) = \zeta(s), \quad (Re(s) > 1) \tag{38}$$

# D Integral of the spectral entropy of radiation

Using Equation 21 the integral is reduced to three simpler integrals:

$$\int S_\lambda d\lambda = \int \frac{2hc^2}{T\lambda^5} d\lambda + \int \frac{1}{T} L_\lambda d\lambda + \int \frac{2kc}{\lambda^4} \cdot \log\left(\frac{1}{e^{\frac{hc}{\lambda kT}} - 1}\right) d\lambda \tag{39}$$

In the following I will do the change of variable $\frac{hc}{\lambda kT} = x \to -\frac{hc}{\lambda^2 kT} d\lambda = dx$. The three integrals are solved separately, named $i)$, $ii)$ and $iii)$ respectively. Integral $i)$ is reduced to:

$$\int \frac{2hc^2}{T\lambda^5} d\lambda = \int \frac{2hc^2}{T\lambda^5} \frac{-kT}{hc} \lambda^2 dx = \int \frac{-2kc}{\lambda^3} dx = \int -2kc \frac{k^3T^3}{h^3c^3} x^3 dx = \frac{-2k^4T^3}{h^3c^2} \frac{x^4}{4} \tag{40}$$

Integral $ii)$ is the same integral than in the case of the energy. It is solved using the relation in Equation 37 with $k = 3$:

$$\begin{aligned}
\int \frac{1}{T} L_\lambda d\lambda &= \frac{1}{T} \int \frac{2hc^2}{\lambda^5} \frac{1}{e^{\frac{hc}{\lambda kT}} - 1} d\lambda = \frac{1}{T} \int \frac{2hc^2}{\lambda^5} \frac{1}{e^{\frac{hc}{\lambda kT}} - 1} \frac{-kT}{hc} \lambda^2 dx \\
&= \frac{1}{T} \frac{-2k^4T^4}{h^3c^2} \int x^3 \frac{1}{e^x - 1} dx = \frac{-2k^4T^3}{h^3c^2} \int x^3 \frac{e^{-x}}{1 - e^{-x}} dx \\
&= \frac{-2k^4T^3}{h^3c^2} \int x^3 Li_0(e^{-x}) dx \\
&= \frac{-2k^4T^3}{h^3c^2} \left( -\sum_{n=0}^{k=3} x^{k-n} Li_{n+1}(e^{-x}) \frac{\Gamma(k+1)}{\Gamma(k+1-n)} \right) \\
&= \frac{2k^4T^3}{h^3c^2} \left( x^3 Li_1(e^{-x}) + 3x^2 Li_2(e^{-x}) + 6x Li_3(e^{-x}) + 6 Li_4(e^{-x}) \right)
\end{aligned} \tag{41}$$

Let's solve now integral *iii)*. Although there are many ways to solve it, the simplest way is by making use of the relation between the logarithm and polylogarithms, i.e., using the fact that $Li_1(x) = \sum_{k=1}^{\infty} \frac{x^k}{k} = -\log(1-x)$:

$$
\begin{aligned}
iii) \quad & \int \frac{2kc}{\lambda^4} \log\left(\frac{1}{e^{\frac{hc}{\lambda kT}} - 1}\right) d\lambda = \frac{-2k^4T^3}{h^3c^2} \int x^2 \log\left(\frac{1}{e^x - 1}\right) dx \\
&= \frac{-2k^4T^3}{h^3c^2} \int x^2 \log\left(\frac{e^{-x}}{1-e^{-x}}\right) dx \\
&= \frac{-2k^4T^3}{h^3c^2} \left\{\int x^2 \log\left(e^{-x}\right) dx - \int x^2 \log\left(1-e^{-x}\right) dx\right\} \\
&= \frac{-2k^4T^3}{h^3c^2} \left\{-\frac{x^4}{4} - \int x^2 \left(-Li_1(e^{-x})\right) dx\right\}
\end{aligned} \tag{42}
$$

The latest integral can solved doing integration by parts and knowing the properties of the polylogarithms. In particular, I will use the derivative property (Eq. 32), $\frac{d}{dx}\left[Li_1(e^{-x})\right] = -Li_0(e^{-x})$, and the same summation relation used before (Eq. 37):

$$
\begin{aligned}
iii) \quad & \frac{-2k^4T^3}{h^3c^2} \left\{-\frac{x^4}{4} + \int x^2 Li_1(e^{-x}) dx\right\} \\
&= \frac{-2k^4T^3}{h^3c^2} \left\{-\frac{x^4}{4} + \frac{x^3}{3} Li_1(e^{-x}) - \int \frac{x^3}{3} \left(-Li_0(e^{-x})\right) dx\right\} \\
&= \frac{-2k^4T^3}{h^3c^2} \left\{-\frac{x^4}{4} + \frac{x^3}{3} Li_1(e^{-x}) + \frac{1}{3}\left(-\sum_{n=0}^{k=3} x^{k-n} Li_{n+1}(e^{-x}) \frac{\Gamma(k+1)}{\Gamma(k+1-n)}\right)\right\}
\end{aligned} \tag{43}
$$

Once the three integrals are completed, they can be put together, which reduces to (note: the sum expression is typed as: $\left(-\sum_{n=0}^{k=3} \ldots\right) = -\{x^3 Li_1(e^{-x}) + 3x^2 Li_2(e^{-x})$ $+6x Li_3(e^{-x}) + 6Li_4(e^{-x})\}$ ):

$$
\begin{aligned}
\int S_x dx &= \frac{-2k^4T^3}{h^3c^2} \left\{\frac{x^4}{4} + \left(-\sum_{n=0}^{k=3} \ldots\right) - \frac{x^4}{4} + \frac{x^3}{3} Li_1(e^{-x}) + \frac{1}{3}\left(-\sum_{n=0}^{k=3} \ldots\right)\right\} \\
&= \frac{-2k^4T^3}{h^3c^2} \left\{\frac{x^3}{3} Li_1(e^{-x}) + \frac{4}{3}\left(-\sum_{n=0}^{k=3} \ldots\right)\right\} \\
&= \frac{-2k^4T^3}{h^3c^2} \left\{\frac{x^3}{3} Li_1(e^{-x}) - \frac{4}{3}\left(x^3 Li_1(e^{-x}) + 3x^2 Li_2(e^{-x}) + 6x Li_3(e^{-x}) + 6Li_4(e^{-x})\right)\right\} \\
&= \frac{2k^4T^3}{h^3c^2} \left\{x^3 Li_1(e^{-x}) + 4x^2 Li_2(e^{-x}) + 8x Li_3(e^{-x}) + 8Li_4(e^{-x})\right\}
\end{aligned} \tag{44}
$$

For isotropic radiation (radiant intensity is independent of direction), the monochromatic flux density is $F_\lambda = \pi\ L_\lambda$, and the same accounts for the entropy. Multiplying the previous equation by $\pi$, and using the Stefan's constant $\sigma = \dfrac{2\pi^5 k^4}{15 h^3 c^2}$:

$$\Im_S = \frac{15}{\pi^4}\sigma T^3 \left\{x^3 Li_1(e^{-x}) + 4x^2 Li_2(e^{-x}) + 8x Li_3(e^{-x}) + 8 Li_4(e^{-x})\right\} \tag{45}$$

When the whole spectrum is considered, the polylogarithmic term is reduced to $8\zeta(8) = \frac{4\pi^4}{45}$, and the obtained entropy flux density is:

$$\Im_S = \frac{15}{\pi^4}\sigma T^3 \left(\frac{4\pi^4}{45}\right) = \frac{4}{3}\sigma T^3 \tag{46}$$

in agreement with the thermodynamic theory [6].

# E Mean of the energy and entropy of radiation

The Mean of the normalized distribution in the $x$ variable is determined as:

$$\int x \cdot S_x dx = \frac{45}{4\pi^4}\left\{\int x^4 dx + \int x^4 \frac{1}{e^x - 1} dx + \int x^3 \log\left(\frac{1}{e^x - 1}\right) dx\right\} \tag{47}$$

In order to obtain the value of the integral, it will be divided into two parts. The first part, $\displaystyle\int x^4 \frac{1}{e^x - 1} dx$ is the one with elements similar to the energy and will be named $i$), and the second part will be the rest of it, named $ii$). Although one can think that it would be simpler to do each integral separately, the third integral (the one with the logarithm) is not convergent and cannot be solved alone. However, the introduction of the $x^4$ factor reduces its complexity and makes it integrable, as it will be seen below.

The integral of the first part, i.e. the energy term $i$), is called Bose-Einstein integral, and its expression is, in general:

$$I(p) = \int_0^\infty \frac{x^{p-1}}{e^x - 1} dx \tag{48}$$

Using series expansion:

$$\frac{1}{1 - e^{-x}} = \sum_{k=0}^{\infty} (e^{-x})^k \tag{49}$$

the integral is:

$$\int_0^\infty \frac{x^{p-1}}{e^x-1}dx = \int_0^\infty e^{-x}\frac{1}{(1-e^{-x})}x^{p-1}dx = \int_0^\infty e^{-x}\left[\sum_{k=0}^{\infty}(e^{-x})^k\right]x^{p-1}dx$$
$$= \sum_{k=0}^{\infty}\int_0^\infty e^{-(k+1)x}x^{p-1}dx \tag{50}$$

Doing the change of variable:

$$(k+1)x = y \quad \rightarrow \quad dx = \frac{dy}{k+1} \tag{51}$$

the integral is reduced to:

$$\sum_{k=0}^{\infty}\int_0^\infty e^{-(k+1)x}x^{p-1}dx = \sum_{k=0}^{\infty}\frac{1}{(k+1)^p}\int_0^\infty y^{p-1}e^{-y}dy = \Gamma(p)\sum_{k=1}^{\infty}\frac{1}{(k)^p} = \Gamma(p)\zeta(p) \tag{52}$$

where $\Gamma(p)$ is the Euler gamma function and $\zeta(p)$ is the Riemann zeta function. The reader should notice the change in the $k$ index in the final steps of the previous equation.

In this particular case $p = 5$, and the Bose-Einstein integral is then reduced to $\Gamma(5)\zeta(5)$. Therefore:

$$i) \quad \frac{45}{4\pi^4}\int_0^\infty x^4\frac{1}{e^x-1}dx = \frac{45}{4\pi^4}\left[24\cdot\zeta(5)\right] \tag{53}$$

This result is also applicable to the case of the energy, although the pre-factor would be $15/\pi^4$ instead of $45/4\pi^4$. The Mean of the energy of radiation in the $x$ variable is:

$$L_{mean} = \frac{15}{\pi^4}\left[24\cdot\zeta(5)\right] \simeq 3.83223 \tag{54}$$

which in the $\lambda T$ variable is $\simeq 3.75447$ ($\times\ 10^6$ nm K).

Continuing with the entropy, the other terms, i.e. integral *ii*), are integrated like in the previous appendix, using the properties of the polylogarithms:

$$ii) \quad \frac{45}{4\pi^4}\int\left\{x^4 + x^3\log\left(\frac{1}{e^x-1}\right)\right\}dx = \frac{45}{4\pi^4}\int\left\{x^4 + x^3\log\left(\frac{e^{-x}}{1-e^{-x}}\right)\right\}dx$$
$$= \frac{45}{4\pi^4}\int\left\{x^4 + x^3\log\left(e^{-x}\right) - x^3\log\left(1-e^{-x}\right)\right\}dx$$
$$= \frac{45}{4\pi^4}\int\left\{x^4 + x^3(-x) - x^3\log\left(1-e^{-x}\right)\right\}dx = \frac{45}{4\pi^4}\int\left\{x^3 Li_1(e^{-x})\right\}dx$$
$$= \frac{45}{4\pi^4}\left\{\frac{x^4}{4}Li_1(e^{-x}) - \int\frac{x^4}{4}(-Li_0(e^{-x}))dx\right\}$$

$$= \frac{45}{4\pi^4}\left\{\frac{x^4}{4}Li_1(e^{-x}) - \frac{1}{4}\left(\sum_{n=0}^{k=4} x^{4-n} Li_{n+1}(e^{-x})\frac{\Gamma(4+1)}{\Gamma(4+1-n)}\right)\right\}$$
$$= \frac{45}{4\pi^4}\left\{-x^3 Li_2(e^{-x}) - 3x^2 Li_3(e^{-x}) - 6x Li_4(e^{-x}) - 6Li_5(e^{-x})\right\} \quad (55)$$

With the intention to evaluate the solution in 0 and $\infty$, it is useful to know some properties of the polylogarithms. For the argument equal to unity, the polylogarithm is reduced to the Riemann Zeta function, $Li_s(1) = \zeta(s)$ (Equation 38). In this case, when the value of $x$ is zero (or equivalently, the product $\lambda T$ is equal to $\infty$), the argument is $e^{-x} = e^{-0} = 1$.

On the other hand, using L'Hôpital rule recursively it can be proved that [19]:

$$\lim_{x\to\infty} x^{4-n} Li_{n+1}(e^{-x}) = 0, \text{ n = 0,1,2,3,4} \quad (56)$$

With all this, equation $ii)$ evaluated in $[0,\infty)$ is reduced to:

$$ii) \quad \frac{45}{4\pi^4}\int\left\{x^4 + x^3\log\left(\frac{1}{e^x-1}\right)\right\}dx = \frac{45}{4\pi^4}\left[6Li_5(e^0)\right] = \frac{45}{4\pi^4}\left[6\zeta(5)\right] \quad (57)$$

Thus, the total value of the Mean in the $x$ variable is $i) + ii)$:

$$\int_0^\infty x\cdot S_x dx = \frac{45}{4\pi^4}\left[6\zeta(5) + 24\zeta(5)\right] = \frac{45}{4\pi^4}\left[30\zeta(5)\right] \simeq 3.59272 \quad (58)$$

In the $\lambda T$ variable, the Mean is $\simeq 4.00477$ ($\times 10^6$ nm K).

# F Higher moments: Variance, Skewness and Kurtosis

In this section, I will calculate the moments of the distribution until the fourth order, following the very same formalism than in the previous appendix.

The moment of order one corresponds to the Mean, which has been calculated before:

$$E[x] = \frac{45}{4\pi^4}\cdot 30\cdot\zeta(5) \simeq 3.59272 \quad (59)$$

The moment of order two is calculated as:

$$E[x^2] = \int x^2\cdot S_x dx = \frac{45}{4\pi^4}\left\{\int x^5 dx + \int x^5\frac{1}{e^x-1}dx + \int x^4\log\left(\frac{1}{e^x-1}\right)dx\right\} \quad (60)$$

Doing the splitting of the integrals as we did in Appendix E, the order of the Bose-Einstein integral is now $p = 6$, being $i) \int_0^\infty x^5\frac{1}{e^x-1}dx = \Gamma(6)\zeta(6) = 120\zeta(6)$.

The rest of the integral is reduced, using the properties of polylogarithms applied in Eq. 55, to:

$$ii) \quad \frac{45}{4\pi^4}\left\{-x^4 Li_2(e^{-x}) - 4x^3 Li_3(e^{-x}) - 12x^2 Li_4(e^{-x}) - 24x Li_5(e^{-x}) - 24 Li_6(e^{-x})\right\} \tag{61}$$

Reasoning as before, in the $[0,\infty)$ interval the integral is evaluated as $ii)$ $24\zeta(6)$, and the moment $E[x^2] = i) + ii)$ is:

$$E[x^2] = \frac{45}{4\pi^4}\cdot[120\zeta(6) + 24\zeta(6)] = \frac{45}{4\pi^4}\cdot 144\cdot\zeta(6) \simeq 16.9193 \tag{62}$$

The order three is similarly calculated as:

$$E[x^3] = \int x^3\cdot S_x dx = \frac{45}{4\pi^4}\left\{\int x^6 dx + \int x^6 \frac{1}{e^x - 1}dx + \int x^5 \log\left(\frac{1}{e^x-1}\right)dx\right\} \tag{63}$$

where now the Bose-Einstein integral is $\Gamma(7)\zeta(7) = 720\zeta(7)$, and the rest of the integral is reduced to:

$$\begin{aligned} ii) \quad \frac{45}{4\pi^4}\{&-x^5 Li_2(e^{-x}) - 5x^4 Li_3(e^{-x}) - 20x^3 Li_4(e^{-x}) - 60x^2 Li_5(e^{-x}) \\ &-120x Li_6(e^{-x}) - 120 Li_7(e^{-x})\}\end{aligned} \tag{64}$$

and therefore:

$$E[x^3] = \frac{45}{4\pi^4}\cdot[720\zeta(7) + 120\zeta(7)] = \frac{45}{4\pi^4}\cdot 840\cdot\zeta(7) \simeq 97.8235 \tag{65}$$

Finally, for the moment of order 4, the Bose-Einstein integral is $\Gamma(8)\zeta(8) = 5040\zeta(7)$ and the rest of the integral is:

$$\begin{aligned} ii) \quad \frac{45}{4\pi^4}\{&-x^6 Li_2(e^{-x}) - 6x^5 Li_3(e^{-x}) - 30x^4 Li_4(e^{-x}) - 120x^3 Li_5(e^{-x}) \\ &-360x^2 Li_6(e^{-x}) - 720x Li_7(e^{-x}) - 720 Li_8(e^{-x})\}\end{aligned} \tag{66}$$

which gives a final value of:

$$E[x^4] = \frac{45}{4\pi^4}\cdot[5040\zeta(8) + 720\zeta(8)] = \frac{45}{4\pi^4}\cdot 5760\cdot\zeta(8) \simeq 667.948 \tag{67}$$

In general, the moment of order $n$ can be determined as:

$$E[x^n] = \frac{45}{4\pi^4}\left[\frac{4+n}{4+n-1}\Gamma(4+n)\zeta(4+n)\right], \qquad n \geq 1 \tag{68}$$

# References

[1] Wien, W. Temperatur und entropie der strahlung. *Annalen der Physik* **288**, 132–165 (1894).

[2] Planck, M. Über eine Verbesserung der Wienschen Spektral Gleichung. *Verhandl. Dtsch. phys. Ges.* **2** (1900).

[3] Planck, M. On the Theory of the Energy Distribution Law of the Normal Spectrum. *Verhandl. Dtsch. phys. Ges.* **2** (1900).

[4] Wien, W. Ueber die energievertheilung im emissionsspectrum eines schwarzen körpers. *Annalen der Physik* **294**, 662–669 (1896).

[5] Rayleigh, L. LIII. Remarks upon the law of complete radiation. *Philosophical Magazine. Series 5* **49**, 539 (1900).

[6] Planck, M. *The Theory of Heat radiation* (P. Blakiston's Son & Co., Philadelphia, 1913), 1 edn.

[7] Einstein, A. Über einen die erzeugung und verwandlung des lichtes betreffenden heuristischen gesichtspunkt. *Annalen der Physik* **322**, 132–148 (1905).

[8] Planck, M. Ueber das Gesetz der Energieverteilung im Normalspectrum. *Annalen der Physik* **309**, 553–563 (1901).

[9] Bose, S. Plancks gesetz und lichtquantenhypothese. *Zeitschrift für Physik* **26**, 178–181 (1924).

[10] Rosen, P. Entropy of radiation. *Physical Review* **96**, 555 (1954).

[11] Laue, M. Die entropie von partiell kohrenten strahlenbndeln. *Annalen der Physik* **328**, 1–43 (1907).

[12] Lorentz, H. The Theory of Radiation and the Second Law of Thermodynamics. *KNAW, Proceedings* **3**, 436–450 (1901).

[13] Kastler, A. Radiation and entropy, coherence and negentropy. In Haken, H. (ed.) *Synergetics*, vol. 2 of *Springer Series in Synergetics*, 18–25 (Springer Berlin Heidelberg, 1977).

[14] Valluri, S. R., Jeffrey, D. J. & Corless, R. M. Some applications of the Lambert W function to physics. *Canadian Journal of Physics* **78**, 823–831 (2000).

[15] Corless, R. M., Gonnet, G. H., Hare, D. E. G., Jeffrey, D. J. & Knuth, D. E. On the Lambert W function. *Advances in Computational Mathematics* **5**, 329–359.

[16] Olver, F. W. J., Lozier, D. W., Boisvert, R. F. & Clark, C. W. *NIST handbook of mathematical functions* (Cambridge University Press, Cambridge, 2010), 1 edn.

[17] Apostol, T. M. Polylogarithm. In Olver, F. W. J., Lozier, D. M., Boisvert, R. F. & Clark, C. W. (eds.) *NIST Handbook of Mathematical Functions* (Cambridge University Press, 2010).

[18] Abramowitz, M. & Stegun, I. A. *Handbook of Mathematical Functions with Formulas, Graphs, and Mathematical Tables* (Dover Publications, New York, 1972).

[19] Stewart, S. A. M. Blackbody radiation functions and polylogarithms. *Journal of Quantitative Spectroscopy & Radiative Transfer* **113**, 232 – 238 (2012).
